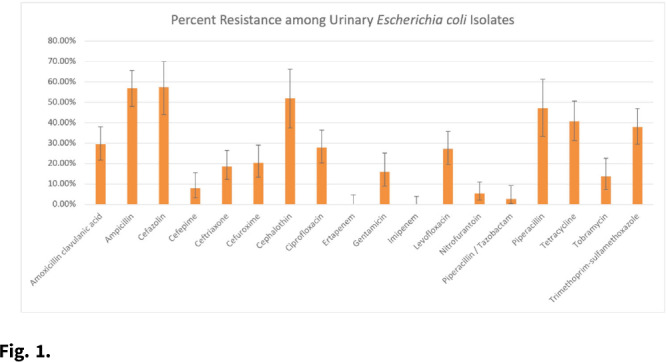# Prior cultures predict subsequent susceptibility in patients with recurrent urinary tract infections

**DOI:** 10.1017/ash.2022.187

**Published:** 2022-05-16

**Authors:** Marissa Valentine-King, Barbara Trautner, Roger Zoorob, George Germanos, Jason Salemi, Kalpana Gupta, Larissa Grigoryan

## Abstract

**Background:** Patients with recurrent urinary tract infections (rUTI) experience frequent exposure to antimicrobial regimens, leaving them at higher risk for developing antibiotic resistance. Little information on the prevalence of antibiotic resistance among patients with rUTI has been published. Although the IDSA recommends using a prior culture to guide empiric treatment, studies have not examined the predictive ability of a prior culture among patients meeting rUTI criteria. We constructed an antibiogram and evaluated test metrics, including sensitivity, specificity, and positive predictive value (PPV) and negative predictive values (NPV) of a prior culture (any organism), on predicting resistance (PPV) or susceptibility (NPV) of a future culture among patients with uncomplicated rUTI in an outpatient setting. **Methods:** We retrospectively extracted electronic health record data from outpatients aged ≥18 years who had an ICD-10 code for cystitis listed twice in 6 months or thrice in 12 months between November 1, 2016, and December 31, 2018. Patients sought care at either urology or primary care practices within an academic medical center in Houston, Texas. Patients with functional or structural abnormalities of the genitourinary tract, signs or symptoms of pyelonephritis, or pregnancy were excluded. Antibiogram data were reported for uropathogens with ≥30 isolates, and intermediate results were considered resistant. Test metrics and Bayes’ PPV and NPV were calculated using standard formulas. **Results:** We included 597 visits from 232 unique patients. Most were White (63%) and female (92%), and the cohort had a median age of 58 (IQR, 41–68). Among 310 rUTI episodes with a urine culture, 189 (61%) had at least 1 uropathogen isolated, and *Escherichia coli* (n = 130, 66%) was most common among all 196 uropathogens. *E. coli* isolates had >20% resistance to 10 of 18 antibiotics (Fig. 1). *E. coli* resistance to ciprofloxacin was 27.9%, resistance to nitrofurantoin was 5.5%, and resistance to trimethoprim-sulfamethoxazole was 38.0%. The PPVs for predicting resistance were highest for ceftriaxone (0.86; 95% CI, 0.60–0.96), ciprofloxacin (0.84; 95% CI, 0.63–0.94), and levofloxacin (0.84; 95% CI, 0.63–0.94). NPVs of resistance were highest for gentamicin (0.97; 95% CI, 0.83–1.00), ceftriaxone (0.94; 95% CI, 0.86–0.98), and cefepime (0.94; 95% CI, 0.84–0.98), whereas NPVs for cefuroxime, ciprofloxacin, levofloxacin, and nitrofurantoin were all >0.83. **Conclusions:** We detected considerable antibiotic resistance among patients with rUTI to commonly prescribed antibiotics. Prior urine culture susceptibility demonstrated moderate-to-high PPVs for predicting future resistance to ceftriaxone and fluoroquinolones as well as high NPVs for several cephalosporins and fluoroquinolones, which could inform empiric prescribing choices.

**Funding:** This investigator-initiated research study was funded by Rebiotix, a Ferring Company.

**Disclosures:** None

**Figure:**